# Geographical separation and physiology drive differentiation of microbial communities of two discrete populations of the bat *Leptonycteris yerbabuenae*

**DOI:** 10.1002/mbo3.1022

**Published:** 2020-03-17

**Authors:** Osiris Gaona, Daniel Cerqueda‐García, Andrés Moya, Ximena Neri‐Barrios, Luisa I. Falcón

**Affiliations:** ^1^ Posgrado en Ciencias Biológicas de la Universidad Nacional Autonóma de México Instituto de Ecología UNAM Mexico City Mexico; ^2^ Laboratorio de Ecología Bacteriana Instituto de Ecología Universidad Nacional Autonóma de México UNAM Parque Científico y Tecnológico de Yucatán Mérida Mexico; ^3^ Consorcio de Investigación del Golfo de México (CIGOM) Centro de Investigación y de Estudios Avanzados del Instituto Politécnico Nacional Unidad Mérida, Departamento de Recursos del Mar Mérida Mexico; ^4^ Instituto de Biología Integrativa de Sistemas Universidad de Valencia y Consejo Superior de Investigaciones Científicas (CSIC) Valencia Espana

**Keywords:** geographical separation, holobiont, populations, reproductive stages

## Abstract

In this paper, we explore how two discrete and geographically separated populations of the lesser long‐nosed bat (*Leptonycteris yerbabuenae*)—one in central and the other in the Pacific region of Mexico—differ in their fecal microbiota composition. Considering the microbiota–host as a unity, in which extrinsic (as food availability and geography) or intrinsic factors (as physiology) play an important role in the microbiota composition, we would expect differentiation in the microbiota of two geographically separated populations. The Amplicon Sequences Variants (ASVs) of the V4 region of the 16s rRNA gene from 68 individuals were analyzed using alpha and beta diversity metrics. We obtained a total of 11 566 (ASVs). The bacterial communities in the Central and Pacific populations had a diversity of 6,939 and 4,088 ASVs, respectively, sharing a core microbiota of 539 ASVs accounting for 75% of the relative abundance, suggesting stability over evolutionary time. The Weighted UniFrac metrics tested by a PERMANOVA showed that lactating and pregnant females had significant beta diversity differences in the two populations compared with other reproductive stages. This could be a consequence of the increased energy requirements of these physiological stages, more than the variation due to geographical separation. In contrast, a positive correlation of the observed ASVs of fecal microbiota with the observed ASVs of plastids related to the diet was observed in the juveniles and adults, suggesting that in these physiological stages an extrinsic factor as the diet shapes the microbiota composition. The results provide a baseline for future studies of the microbiome in these two wild populations of the lesser long‐nosed bat, the main pollinator of the Agaves from which the beverages tequila and mezcal are made.

## Graphical abstract

*Leptonycteris yerbabuenae* is a bat species found in Mexico in two separate populations, one in the Pacific and the other in the Central region. With the analysis of its fecal microbiota, we found differentiation between the two populations. However, these populations share a core microbiota of 359 Amplicon Sequences Variants, accounting for 75% of abundance, and suggesting stability over time. The microbiota differs between reproductive stages within the populations, suggesting that factors such as geographic separation and physiological variation drive changes in the microbiota.

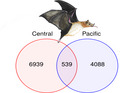

## 1 INTRODUCTION

The microbial communities that inhabit the guts of mammals are complex, dynamic, and critical to the health of the host (Ingala, Becker, Bak Holm, Kristiansen, & Simmons, 2019; Ochman et al., 2010). Microbiota composition and abundance are further determined by several factors such as diet, geography, physiology and health status (Knutie, 2020; Ley et al., 2008; Muegge et al., 2011; Ochman et al., 2010), and even phylogeny. It has been demonstrated, for example, that phylogeny in hominids influences the microbiota at an evolutionary level, suggesting that the microbiota is more than just what the host eats (Ochman et al., 2010).

Microbial symbionts have a variety of roles in the nutrition, immunity, development, reproduction, and speciation of their eukaryote hosts, making this symbiosis a major component of eukaryotic fitness and evolution (Brucker & Bordenstein, 2012). Interactions between host and microbiome have coevolved in mutual adaptations into a superorganism association (MacColl, 2011) and are key to biological adaptations (Brockhurst & Koskella, 2013).

The hologenome concept of evolution states that in addition to the host genome, a significant proportion of the microbiome is also transmitted from one host generation to the next and can thus propagate unique properties of the holobiont (Rosenberg & Zilber‐Rosenberg, 2019). Furthermore, genetic variation can occur by changes in the host and/or microbiome genomes; the microbiome genome is even expected to be able to adjust to environmental dynamics faster and through more processes that the host, and thus may be playing fundamental roles in the adaptation and evolution of holobionts (Rosenberg & Zilber‐Rosenberg, 2019).

Genetic variation in holobionts can occur by mutation and DNA rearrangement, amplification or reduction of specific microbes, acquisition of novel microbes from the environment, and horizontal gene transfer from microbe to microbe or from microbe to host (Zilber‐Rosenberg & Rosenberg, 2008). The multilayered structure of the holobiont consists of a core of host‐adapted microbiota assembled from diverse environments and determined by genetic factors, as well as a flexible pool of microbes that depend on environmental diversity and external conditions (Shapira, 2016).

Vertical transmission of the microbiota from parent to progeny allows its maintenance between generations and is likely favored by selection when those microbes are beneficial to the host (Shapira, 2016). The functional profile of the microbiome is probably more constrained by evolution/vertical inheritance than individual bacterial taxa (Martiny et al., 2006; Phillips et al., 2017; Shapira, 2016). Thus, vertical transmission could drive coevolution, resulting in phylogenetic congruence (Shapira, 2016).

The mutualistic and adaptive potential of microbiota offers flexibility and the ability to adapt to a range of ecological niches (Alberdi, Aizpurua, Bohmann, Zepeda‐Mendoza, & Gilbert, 2016). Subsequently, mutualist‐facilitated adaptations can lead to population fragmentation, isolation, and speciation (Shapira, 2016). Speciation—the splitting of a population into two reproductively incompatible populations, each taking a distinct evolutionary path—is a defining process in evolution. Mutualist microbes can drive all modes of isolation, including ecological, behavioral, and developmental asynchronization and genetic divergence, but, as facilitators of niche adaptation, they are frequently associated with ecological isolation (Brucker & Bordenstein, 2012; Shapira, 2016).

Early evidence suggested that in the order Chiroptera, microbiome composition was influenced by the host phylogeny and life history (Phillips et al., 2012). In contrast, recent studies find no evidence of phylosymbiosis in Afrotropical bats (Lutz et al., 2019). Due to their species‐specific feeding strategy specialization, Phyllostomid bats could remain as a model clade to study the relationship of the microbiome–host composition, phylogeny, and coevolution (Carrillo‐Araujo et al., 2015). Phyllostomid bats are found from the southern USA and northern Mexico to Argentina and show a great evolutionary diversification of species, in which patterns are dependent on geographical and ecological interactions, resulting in a great diversity of dietary strategies and the most ecologically diverse family within the order Chiroptera (Carrillo‐Araujo et al., 2015). They show a remarkable degree of evolutionary diversification of dietary strategies, from insectivory (the ancestral trait) (Monteiro & Nogueira, 2011) to feeding on blood, small vertebrates, nectar, fruit, and complex omnivorous diets (Gardner, 1976).

*Leptonycteris yerbabuenae* is a migratory phyllostomid bat. It is usually a strict and specialized nectar feeder, though it can occasionally consume fruit, so its diet is generally poor in proteins and minerals (Fleming & Nassar, 2002). *L. yerbabuenae* feeds mainly on nectar from agaves, columnar cacti like *Carnigea gigantea*, different species of Stenocereus and Bombacaceae (*Psedobombax elipticum*), Convolvulaceae, and other legumes (Arita, 1991; Arita & Humphrey, 1988; Cole & Wilson, 2006; Valiente‐Banuet, Arizmendi, Rojas‐Martínez, & Domínguez‐Canseco, 1996).

Mexico has two differentiated populations of *L. yerbabuenae*: one along the Pacific coast including Baja California, Sonora, and Jalisco states, and the other in the south‐central region, including Oaxaca, Morelos, and Guerrero states (Morales‐Garza, Arizmendi, Campos, Martínez‐Garcia, & Valiente‐Banuet, 2007). The two populations are separated geographically, with negligible gene flow, as demonstrated by Morales‐Garza et al., 2007 using random amplified polymorphic DNA (RAPD) analysis (Morales‐Garza et al., 2007).

The population of *L. yerbabuenae* that resides between the latitudes of 3°N and 21°S in North America carry out latitudinal migrations, while populations that reside south of 21°S latitude are year‐round residents. Migrations are known to be correlated with the availability of floral resources (Rojas‐Martínez, Valiente‐Banuet, Coro Arizmendi, Alcántara‐Eguren, & Arita, 1999), of which the most predictable are cacti, agave, and C3 plant nectar (Burke, Frey, Ganguli, & Stoner, 2019). The central population has more genetic variability than the Pacific population, likely due to the environmental stability of central Mexico (Morales‐Garza et al., 2007; Valiente‐Banuet et al., 1996).

In both populations, roosts are differentiated by reproductive stage (Ceballos, Fleming, Chavez, & Nassar, 2006; Cockrum, 1991; Stoner, Salazar, Fernández, & Quesada, 2003), with mating and maternity roosts separated geographically from bachelor and nonreproductive caves (Fleming & Nassar, 2002; Hayward & Cockrum, 1971; Sánchez & Medellín, 2007; Stoner et al.., 2003). Pregnant females congregate in maternity colonies to give birth, lactate, and care for their offspring (Ceballos et al., 2006), and they usually return to the same roost year after year in different stages of pregnancy and offspring rearing throughout their lifetimes (Hayward & Cockrum, 1971). Adult males and nonreproductive females often segregate into groups called “bachelor colonies” (Ceballos et al., 2006). Before foraging at night, both sexes rest in temporary night roosts (Ceballos et al., 2006; Cole & Wilson, 2006).

A study of the fecal microbiota of different reproductive stages within the central population of *L. yerbabuenae* showed that microbiota diversity is related to the reproductive stage rather than geographical distribution within this population (Gaona, Gómez‐Acata, Cerqueda‐García, Neri‐Barrios, & Falcón, 2019). Microbiota composition is consistent in juveniles and nonreproductive females and males, regardless of the roost. Pregnant and lactating females' microbiotas were similar and more diverse than juveniles and nonreproductive adults. One explanation for this is that microbiota evolved with its host to be flexible enough to shift from a specialized diet to a more generalist diet to cope with the increased energy requirements during pregnancy and lactation (Gaona et al., 2019).

The aim of this research is to evaluate the composition of fecal microbiota in two discrete, geographically separated and genetically differentiated lesser long‐nosed bat (*L. yerbabuenae)* populations in Mexico (Morales‐Garza et al., 2007). Our hypothesis is that the geographical separation and reproductive isolation of the two *L. yerbabuenae* populations will lead to significant differences in their fecal microbial composition, making it more similar within populations than between them. We also evaluate whether the heterogeneity within the Pacific population is due to differences between the reproductive stages, as occurs in the central population (Gaona et al., 2019).

## 2 METHODS

### 2.1 Study site

**Figure 1:**
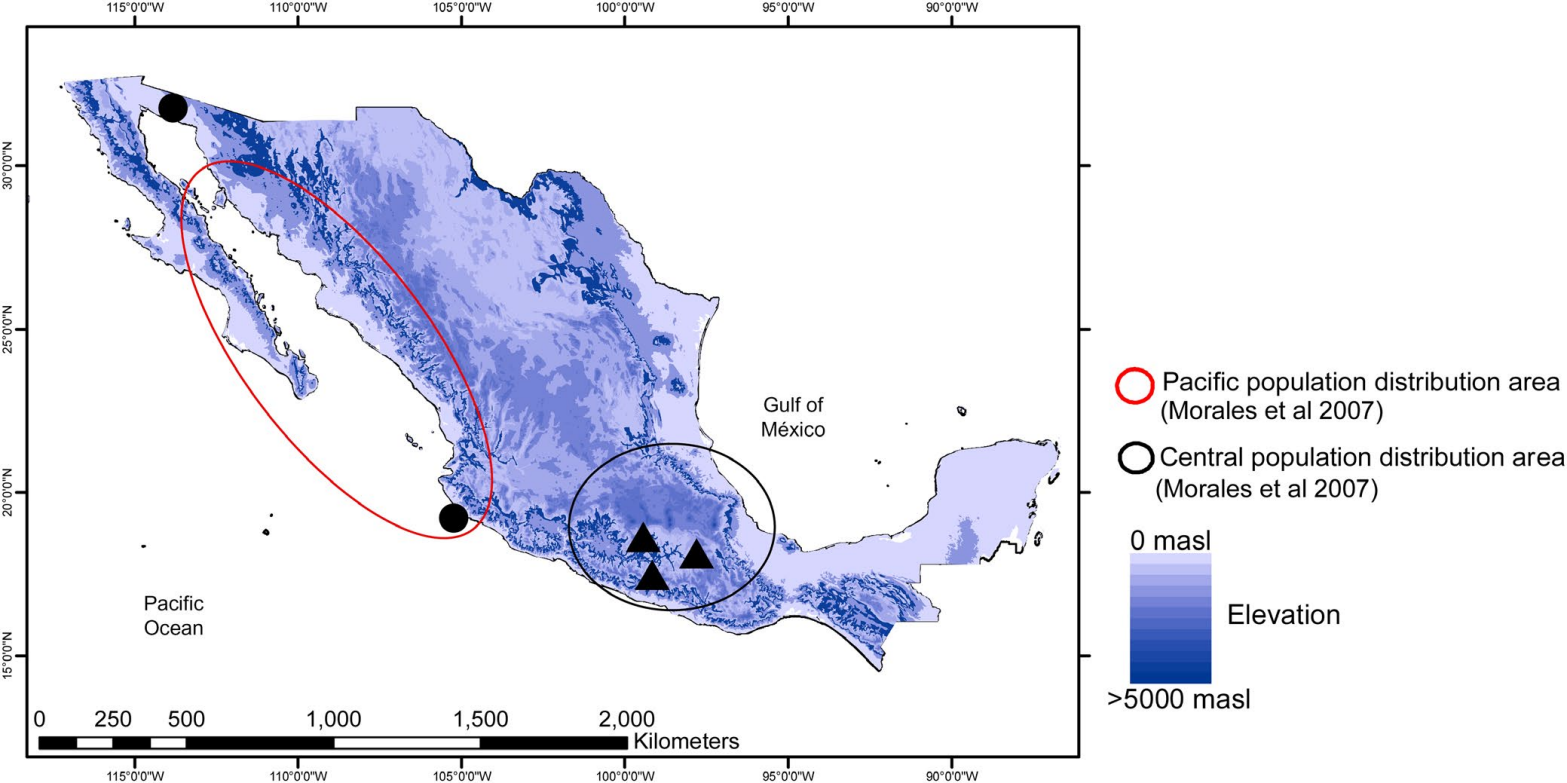
Distribution of *Leptonycteris yerbabuenae* and sampling sites in Mexico

Bat fecal microbiome samples from the lesser long‐nosed bat, *L. yerbabuenae*, were collected at five bat roosts. Two were from the Pacific population—a roost of pregnant and lactating females in Pinacate, Sonora (32°0′0″N, 113°55′0″W), and a roost of reproductive males and juvenile males and females from Panchito cave, Jalisco (98°55′N, 19°32′W) (Figure 1). The remaining three roosts were from the central population: reproductive and juvenile males were sampled in San Juan Noxchitlan, Oaxaca (97°40′N, 18°03′W), a colony of 100,000 bats (Valiente‐Banuet et al., 1996). Pregnant and lactating females were sampled in Juxtlahuaca Cave, Guerrero (17°23″3′N, 99°16″1′W), and juvenile males and females and adult males were sampled in Salitre Cave, Morelos (18°45″0.05′N, 99°11″23.17′W) (Figure 1). All fecal samples were collected between January and November 2015 following the different *L. yerbabuenae* reproductive stages (Table 1) in the two populations.

**Table 1:** Fecal samples collected from the Pacific population (*n* = 31) and Central population (*n* = 51)

Reproductive Stage	Pacific population	Central population
Juvenile females	7	2
Juvenile males	5	8
Adult females	0	11
Adult males	8	12
Pregnant	6	9
Lactating	5	9

### 2.2 Bat fecal microbiome sampling

Bats were captured using 12‐m mist nets (Avinet) at the entrance of the caves using Kunz's technique (Kunz, Betke, Hristov, & Vonhof, 2009) between 18:30 and 7:00 hr. Standard measurements were taken in order to confirm identification and assess the age and reproductive stage of individuals (Anthony, 1988; Kunz, Wemmer, & Hayssen, 1996). All fecal samples were obtained directly from individuals and frozen in sterile Eppendorf tubes with liquid nitrogen Dewar and stored at a −20°C temperature until processed for DNA extraction (see Appendix 2).

### 2.3 Analysis of the sequence data

The paired‐end 2 × 250 reads were processed in QIIME2 (Bolyen et al., 2019). The reads were denoised with the DADA2 (Callahan et al., 2016) plugin to resolve the amplicon sequence variants (ASVs). Both forward and reverse reads were truncated at 200 bp, and chimeric sequences were removed using the “consensus” method. Representative ASV sequences were taxonomically assigned using the “classify‐consensus‐vsearch plugin” with default arguments, using the SILVA 132 database as a reference (Quast et al., 2013). An alignment was performed with the MAFFT algorithm (Katoh, 2002). After masking positional conservations and gap filtering, a phylogeny was built with the FastTree2 algorithm (Price, Dehal, & Arkin, 2010).

**Figure 2:**
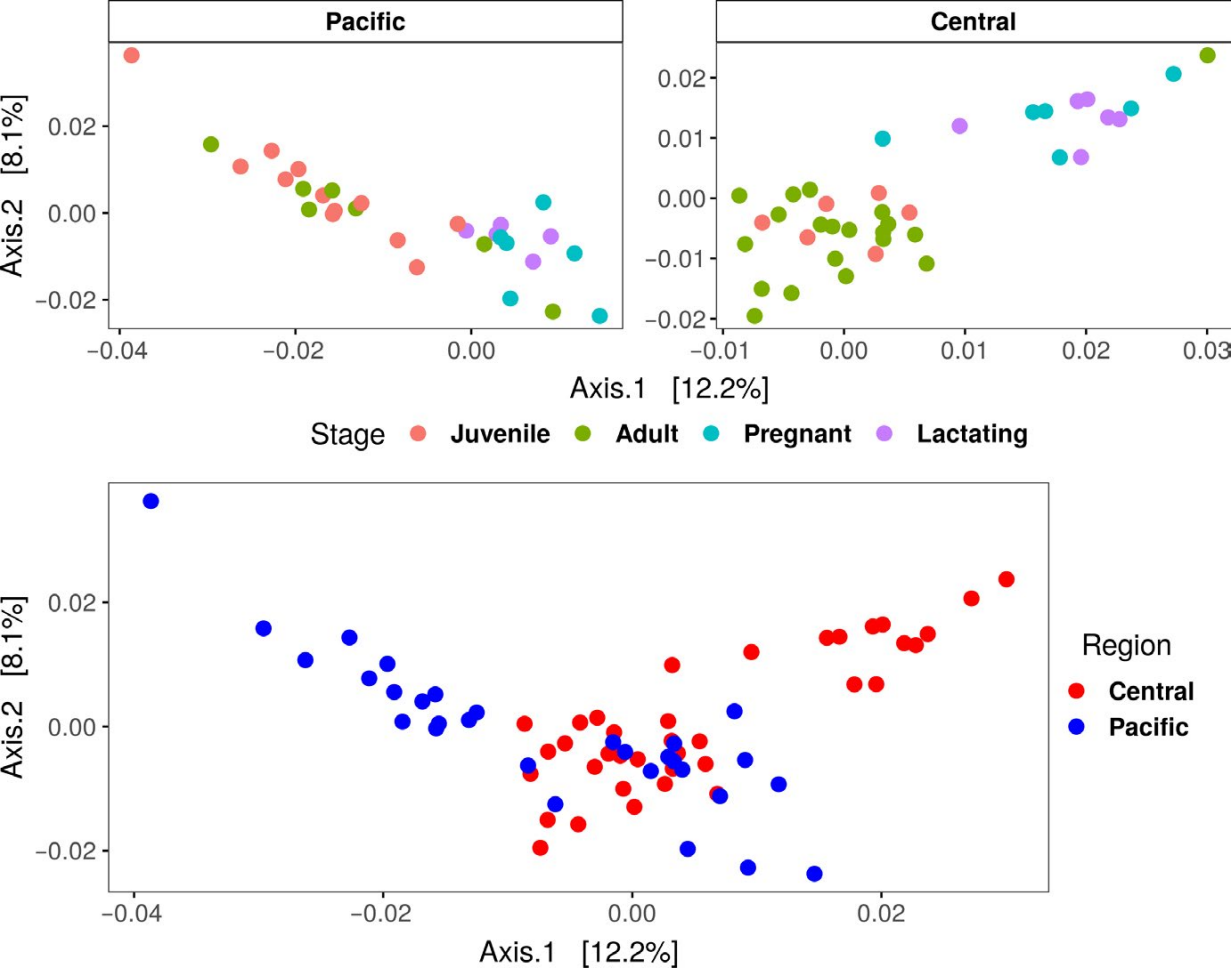
PCoA of weighted UniFrac distance. In the upper panels, each population is shown separately; in the bottom panel, the two populations are shown together

The abundance table and phylogeny were exported to the R environment to perform the statistical analysis with the phyloseq (McMurdie & Holmes, 2013), vegan (Oksanen, 2015), and ggplot2 (Wilkinson, 2011) packages. Plastidic ASVs were filtered out of the samples (for subsequent separate analysis, see below), and then, the samples were rarefied to a minimum sequencing effort of 10,000. A PCoA ordination was performed with the weighted UniFrac distance (Figure 2). The Shannon alpha diversity index was calculated (Figure 4). A PERMANOVA with a weighted UniFrac distance was performed to assess whether there were significant differences among groups of samples (region and reproductive stages as predictor variables) with 1,000 permutations (Table 2).

**Table 2:** PERMANOVA analysis. In the pairwise mode, *p* value was adjusted using the Bonferroni method for multiple comparisons. In the nested mode, stages was nested within the populations in both, for prokaryotes and plastids

Pairwise for prokaryotes	Model *F*	*R^2^*	Adjusted *p* value
Adult versus Juvenile	1.834099	.04090859	.053946054
Adult versus Lactating	2.5512822	.06617892	.005994006*
Adult versus Pregnant	2.7072727	.06818078	.005994006*
Juvenile versus Lactating	3.0078068	.10023414	.005994006*
Juvenile versus Pregnant	3.3985729	.10823973	.005994006*
Lactating versus Pregnant	0.7640991	.03510823	1

	Model *F*	*R^2^*	*p* value
Nested for prokaryote			
Population	3.5636	.04694	.001*
Population:Stage	2.0599	.16279	.001*
Nested for plastids			
Population	19.395	.19493	.001*
Population:Stage	3.350	.20202	.002*

* The significant *p*‐values  < .05.

To explore the differential abundance of taxa, a LEfSe (linear discriminant analysis of effect size) analysis (Segata et al., 2011) was performed at the family level, first with all samples using the populations as categories, and then within each population, using the reproductive stages as categories, using an LDA cutoff >2 and *p* value < .05.

Counts of plastidic ASVs (separated from prokaryotic ASVs before rarefaction) were normalized with the cumulative sum scaling (CSS) method with the metagenomeSeq package (Paulson, Stine, Bravo, & Pop, 2013), and a DPCoA (Double Principle Coordinate Analysis) and the Shannon index were calculated to assess the likely variation in the diet of the bats in the different stages within the regions. A PERMANOVA was carried out with the DPCoA distance matrix to assess the beta diversity between populations. A Pearson correlation analysis was performed with the observed ASVs of plastid and prokaryotic 16S to assess the relationship between microbiota diversity (prokaryotic 16S ASVs) and diet diversity (plastidic ASVs).

## 3 RESULTS

### 3.1 Microbiome composition in the two populations of *Leptonycteris yerbabuenae*

**Figure A1:**
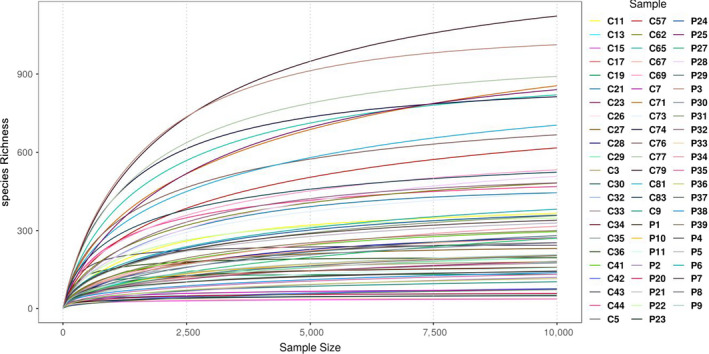
Accumulation curve by simple at 10,000 reads

Of the total samples collected, 68 were positively PCR amplified: 30 for the Pacific population (12 juvenile, 7 adult, 6 pregnant, and 5 lactating) and 38 for the central population (6 juvenile, 20 adult, 6 pregnant, and 6 lactating). We obtained a total of 11 566 ASVs after rarefying to a sampling depth of 10,000 reads (see the accumulation curve in Figure A1 of Appendix 1).

The Principal Coordinates Analysis (PCoA) showed beta diversity difference between the central (Guerrero, Morelos, and Oaxaca) and Pacific (Sonora and Jalisco) *L. yerbabuenae* populations (Figure 2, bottom). Within each population, pregnant and lactating females formed one group and juveniles and nonreproductive adults another (Figure 2. Top left and right).

**Figure 3:**
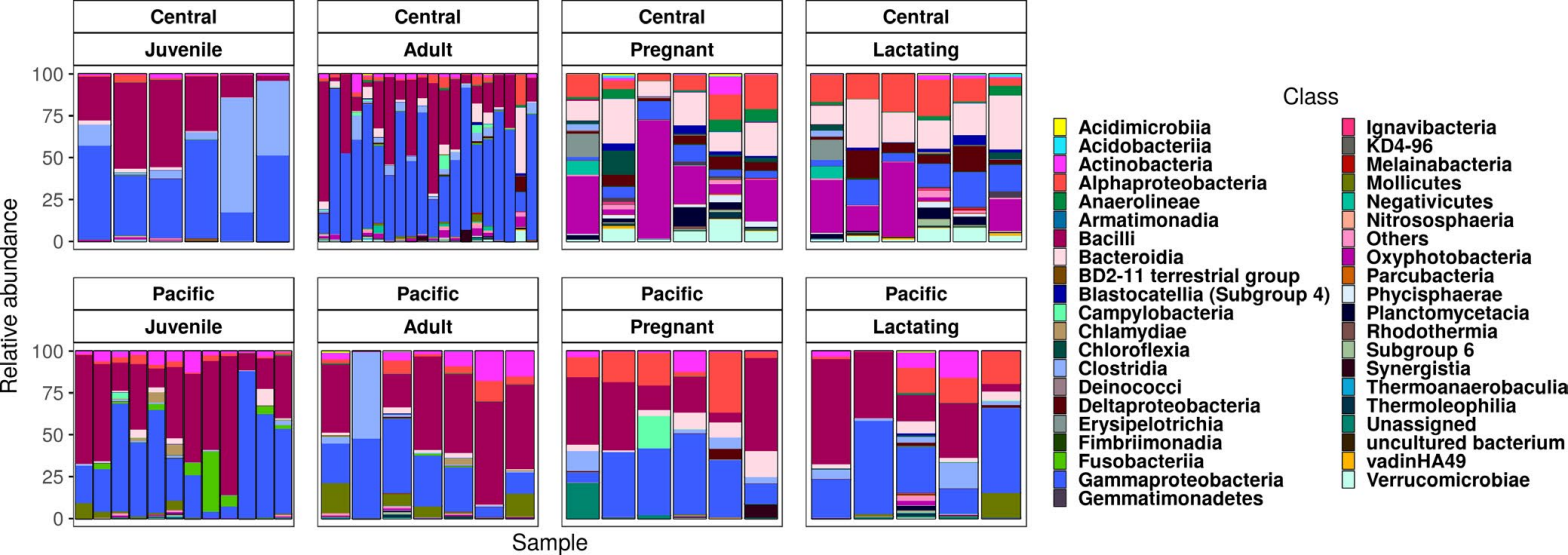
Abundances of the 40 most abundant classes; the remaining classes were grouped in the “others” category

**Figure 4:**
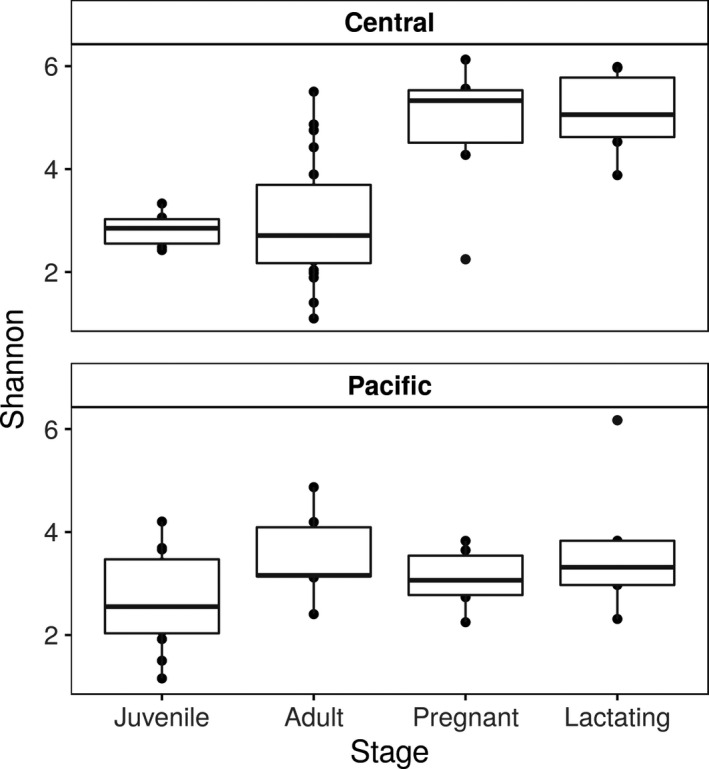
The Shannon index of samples grouped by reproductive stage and population. In the central population, there were significant differences among stages (Kruskal test *p* value < .01, chi‐squared = 14.37206)

Figure 3 shows the 40 most abundant bacterial classes, showing the differences between the composition within each population. There was a trend toward higher diversity among pregnant and lactating females in the central population, but higher diversity among juveniles in the Pacific population (Figures 3 and 4). The Shannon index shows that alpha diversity was more homogeneous within the Pacific population (Figure 4) than in the central population (Gaona et al., 2019).

Comparison using a PERMANOVA test showed differences among reproductive stages; there were significant differences in pairwise comparisons of lactating and pregnant females versus juveniles and adults, and a significant effect of reproductive stage nested within the population (Table 2), showing a clear difference both between the two populations and among stages within each population.

**Figure 5:**
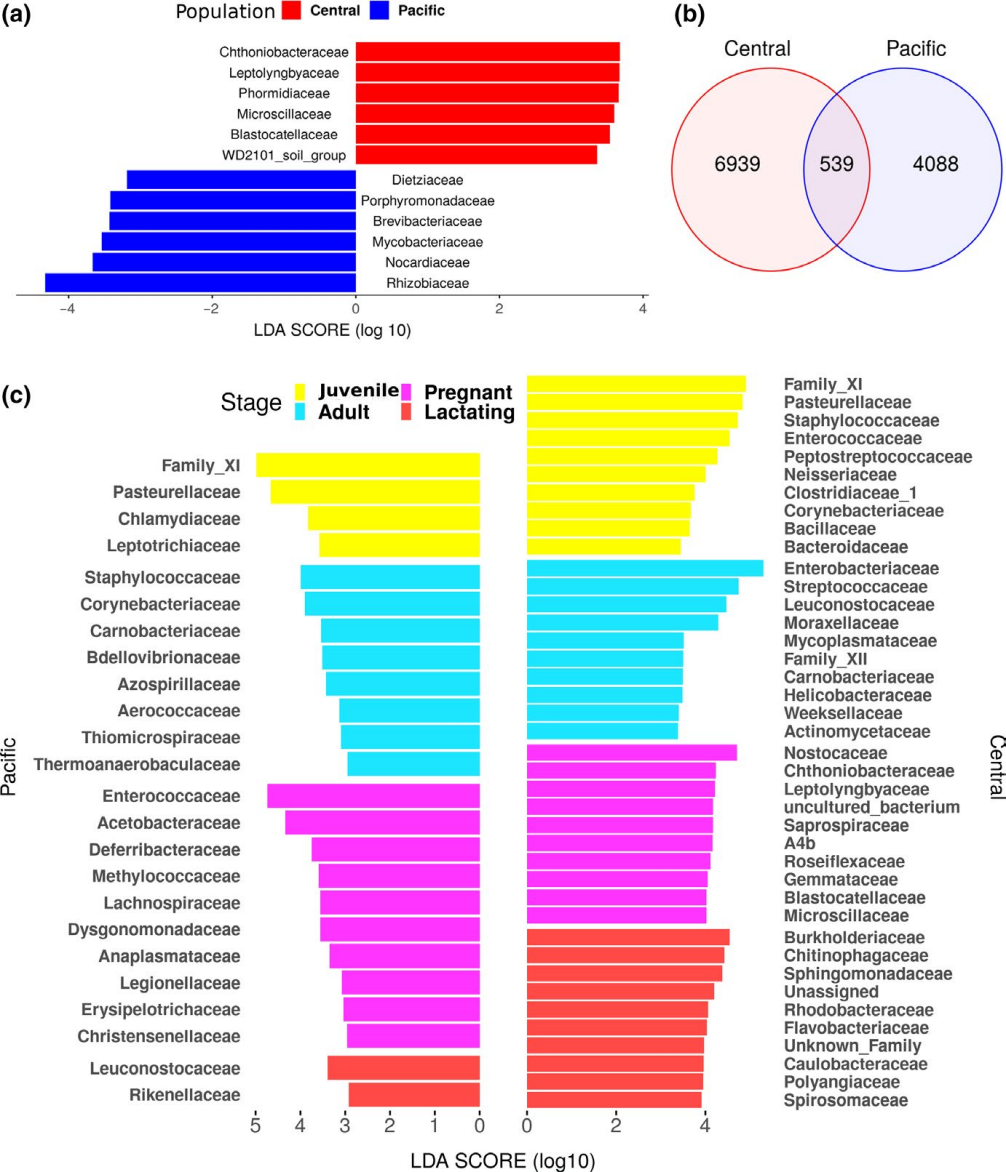
LeFSe analysis of samples with an LDA cutoff > 2 at the family level. (a) Families with differential abundance between populations; (b) Venn diagram showing the ASV shared between populations; (c) Families with differential abundance detected by the LeFSe analysis between reproductive stages within each population—for the central population, just the 10 families with the highest LDA score are shown for each stage (full list provided in Appendix 1)

The LeFSe analysis shows differences among reproductive stages within each population at the family level in bacterial communities (Figure 5a) as well as between the two populations (Figure 5b). The Venn diagram (Figure 5b) shows 539 ASVs that are shared between the two populations. The central population had 6,939 ASVs and the Pacific 4,088 ASVs, a difference of 2,851 ASVs (Figure 5b). The two populations also differed in family‐level abundance. The LefSe analysis identified six differentiated families for each population; in the central population, these were Chtoniobactereaceae, Leptolyngbyaceae, Phormidiaceae, Microscillaceae, and WD2101 soil group; and in the Pacific population, these were Dietziaceae, Porphyromonadaceae, Brevibacteriaceae, Mycobacteriaceae, Nocardiaceae, and Khizobiaceae (Figure 5a).

### 3.2 Bat diet variability between the two regions

**Figure 6:**
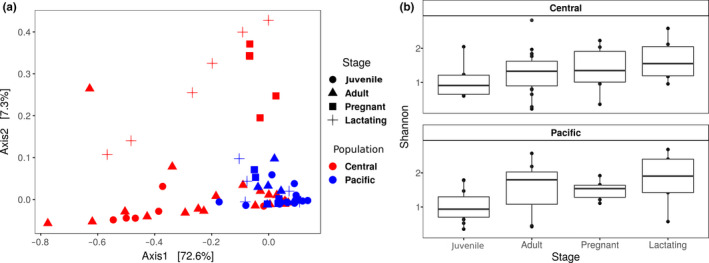
Diversity of plastidic ASVs in each stage within each population. (a) DPCoA ordination showing the distance among the samples; (b) Boxplots of the Shannon index among reproductive stages within each population; there was no significant difference in the Shannon index

The amplification of plastid 16S genes is incidental in fecal microbiome studies. Since chloroplasts are plant organelles acquired by endosymbiosis, their amplification is due to contamination when the host feeds on plant material. Since different plant species’ chloroplasts differ genetically, plastidic 16S gene diversity may provide a relative index of plant diversity in the diet (Knight et al., 2018). The number of plastidic reads in the 68 samples ranged from 17 to 76,979, with a mean of 5,589 (see Table A1). We used the entire plastid dataset with the rationale that although some samples had very low plastid reads, the total reads per sample including bacterial 16S had more than 10,000 reads. After normalization by the CCS method to avoid the effect of differences in the libraries' sample sizes, we calculated the Shannon index and performed a DPCoA and a PERMANOVA (Figure 6 and Table 2).

Similar to the microbiota of the two populations, the beta diversity of chloroplast ASVs was higher in the central population (variance within the population), but alpha diversity was similar (Figure 6). Chloroplast diversity also differed among reproductive stages, with pregnant and lactating females having the highest beta chloroplast diversity (Figure 6).

## 4 DISCUSSION

Changes in microbiome composition could direct speciation, given that the functions of the microbiome have evolutionary consequences in the host (Suzuki, 2017). A central issue in the hologenome concept is whether the hologenome can be considered a unit of selection, in other words, whether the three components of natural selection—variation, differential success, and inheritance—apply to the holobiont (Cerqueda‐García & Falcón, 2016; Moran & Sloan, 2015; Suzuki, 2017). The differences we found between the two populations of *L. yerbabuenae* in Mexico suggest that in this case, there is variation in the holobiont. There is variation between the two populations, shown in the PCoA plot; the diversity within bacterial communities in the central population is larger than in the Pacific population (6,939 ASV, compared with 4,088 ASV), and the beta diversity among groups within the central population is more heterogeneous. In addition, Morales‐Garza et al. (2007) found higher genetic variability in the central population, associated with year‐round availability of pollen and nectar (Fleming & Nassar, 2002; Rojas‐Martínez et al., 1999; Villaseñor, Dávila, & Chiang, 2017). It remains to be tested whether these interpopulation microbiome differences could indicate differential success if the changes in microbial composition are related to losses or gains of metabolic capabilities that contribute to host fitness (Hooper, Littman, & Macpherson, 2012).

**Figure 7:**
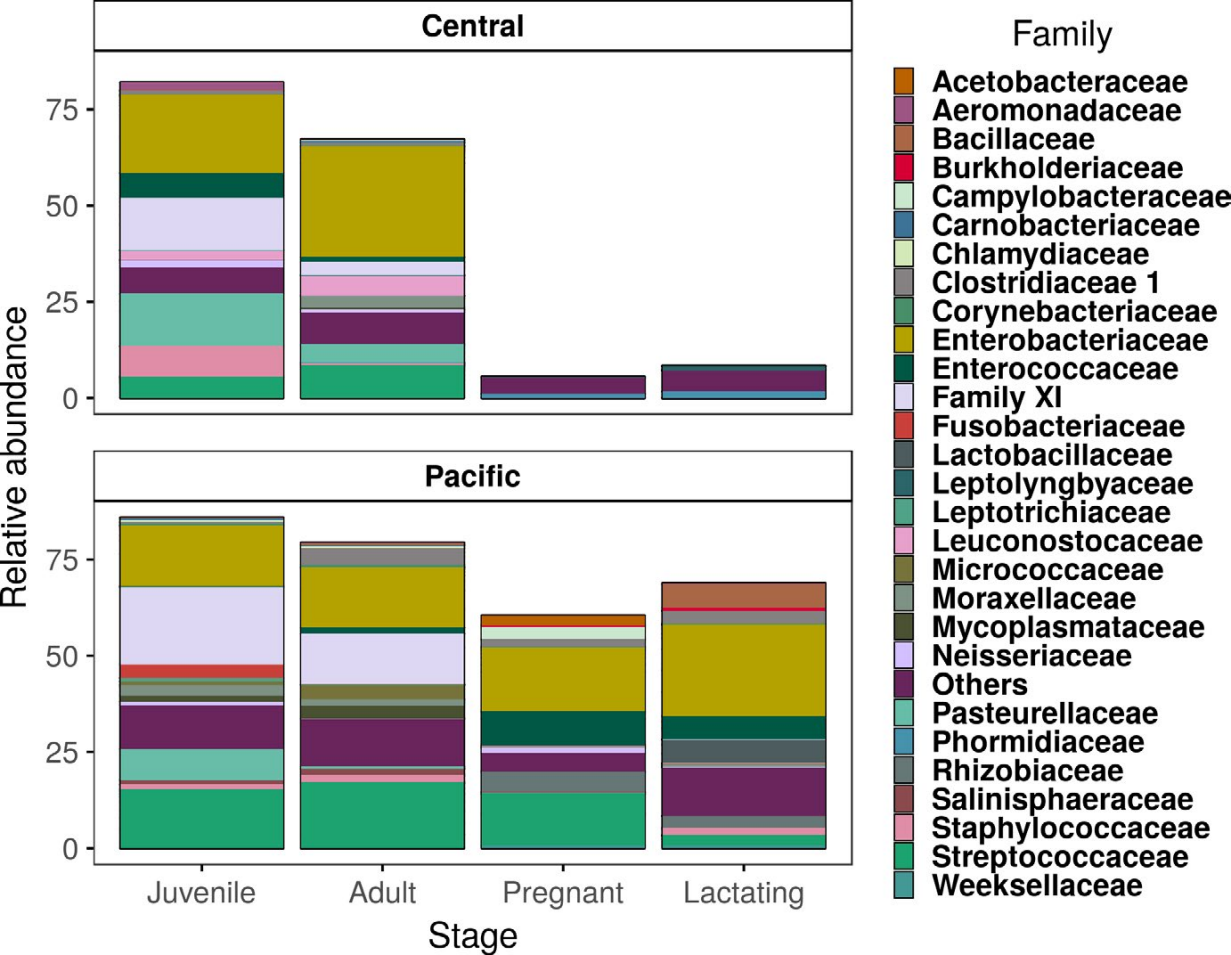
Family classification of the 539 shared ASVs among the two populations. The families with abundance below 1% were combined in the “others” category. The full list of families per sample is shown in Figure A2

**Figure A3:**
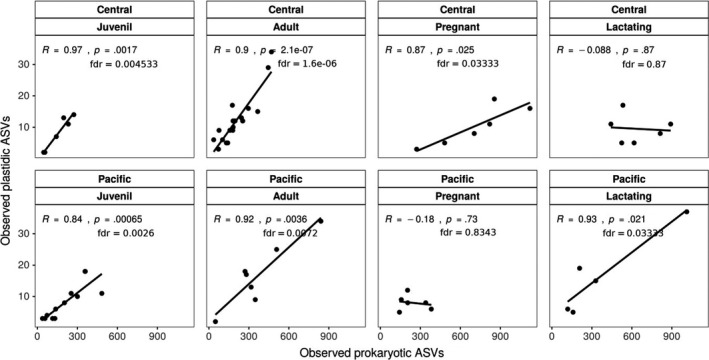
Linear correlation (Pearson), in each stage between prokaryotic and plastid ASVs. The *p* values were adjusted with the FDR method

**Figure A4:**
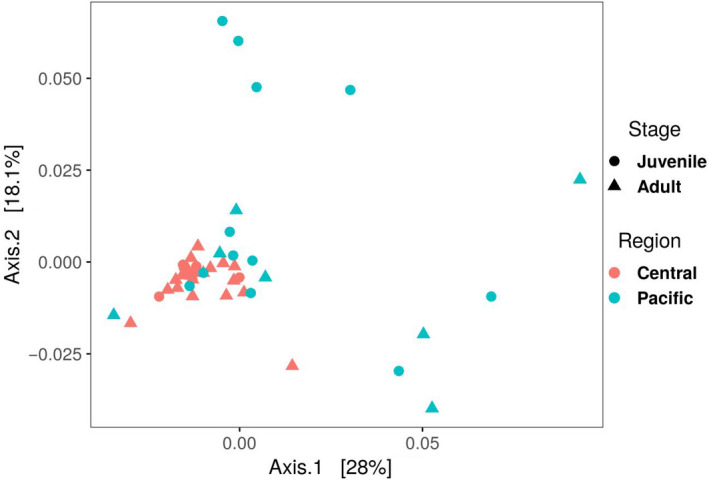
PCoA analysis of juvenile and adult samples with the weighted unifrac distance. The PERMANOVA test showed that central (juvenile‐adult) versus pacific (juvenile‐adult) have significant differences (*F* = 5.262616, R^2^ = .1090412, *p*‐value = .001), with a variance of ~10%

**Figure A5:**
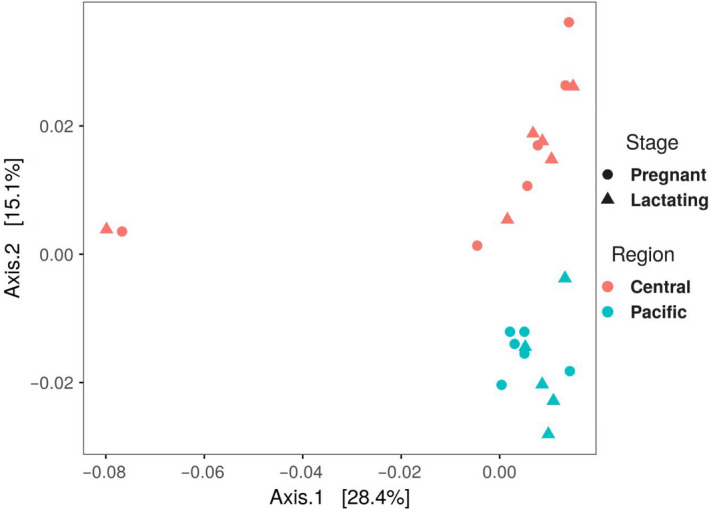
PCoA analysis of pregnant and lactating samples with the weighted unifrac distance. The PERMANOVA test showed that central (pregnant‐lactating) versus pacific (pregnant‐lactating) have significant differences (*F* = 3.1313, *R* ^2^ = .1297609, *p*‐value = .001), with a variance of ~13%

We found that the two populations share a core microbiota of only 539 ASVs and that these are the most abundant bacterial groups for both populations, accounting for about 75% of the relative abundance (Figure 7). This suggests stability in the microbiome within the populations over evolutionary time, and consistent with findings in hominids, the microbiome is more related to the species than to diet (Ochman et al., 2010). Each population has six families with differential abundance detected by the LeFSe analysis (Figure 5a) and a difference of 2,851 ASVs. In addition, ontological characteristics of bats, such as the rearrangement and growth of organs, including the stomach and the enlargement of the intestine, during pregnancy (Speakman, 2008), constrain differences and similarities in the microbiome (Gaona et al., 2019). Despite the changes in beta diversity due to geographical separation, the reproductive stages are more similar between the populations (Figure 3). Flexibility in the microbiome has been confirmed in experiments in which individuals of different species are given a similar diet, their microbiota tends to homogenize (Xiao et al., 2019). Our results partially support this result, since the populations we have studied are more similar within populations. However, we also found a core microbiome that also supports microbiota stability (Coyte, Schluter, & Foster, 2015) and ontogenetic differences in both populations (Gaona et al., 2019). The correlation between microbiota ASVs and plastid ASVs was positive and significant in the adult and juvenile stages (Figure A3), suggesting a more direct diet–microbiota relationship, and thus a Lamarckian acquisition of microbiota. However, this positive correlation is relaxed or broken in the pregnant and lactating stages in both populations. This suggests that in the demanding stages of pregnancy and lactation, the alpha diversity of microbiota increases more steeply than the plastids (diet), suggesting that the physiological state is driving the changes more than the diet. Thus, the core microbiota is present throughout the life cycle, but in pregnancy and lactation, the microbiota is more divergent (Figure 7). The PERMANOVA analysis of only pregnant and lactating females between the two populations showed that these stages have a higher coefficient of variance (*R* ^2^ ~ .13) than the adult and juvenile stages (*R* ^2^ ~ .10) (Figures A4 and A5).

The second and third requirements—differential success and evidence of inheritance of the microbiome—remain to be evaluated. One of the main predictions of microbiome heritability is that the offspring's microbiome will resemble parental microbiome. Thus, a progenitor holobiont (species A with its microbiome) will resemble its holobiont offspring (species B with its microbiome). We are unable to directly evaluate that statement here since we do not have samples of a parental‐progeny holobiont and this study solely focused on one bat species with geographical separation.

The term phylosymbiosis is used to describe the congruence between the differences in bacterial communities and the phylogenetic divergences among species (Brooks, Kohl, Brucker, Opstal, & Bordenstein, 2016; Kohl, Varner, Wilkening, & Dearing, 2018; Mazel et al., 2018), which, in a broad sense, corresponds to the inheritance of the microbiome. While the two populations we sampled in this study are the same species, our data show a relationship between differences in host bacterial communities and genetic differences among populations (Morales‐Garza et al., 2007), which could suggest an early process of phylosymbiosis which has not been explored before in a geographical separation model. As described above, the differences between the two populations of *L. yerbabuenae* show higher variation (beta diversity) in the microbiota the central population (Figures 2 and 4), which also has a higher genetic diversity and is a more stable population (Morales‐Garza et al., 2007).

Macroecological theory indicates that the highest genetic diversity will be found in areas with better access to resources and the best ecological conditions for the species. For this species, the available resources have determined two main divergence routes and very likely the segregation of the species, as confirmed by stable isotope analysis of mitochondrial DNA (Burke et al., 2019), and these changes correspond with orography (Burke et al., 2019). *L. yerbabuenae* are reliant on the availability of flowers, and the central region has year‐long availability (Fleming & Nassar, 2002; Rojas‐Martínez et al., 1999; Villaseñor et al., 2017) in contrast to the seasonal availability of the Pacific region (Petit, Excoffier, & Mayer, 1999). Thus, the reproductive peaks of bats and the population composition are different between the populations, apparently supported by the beta diversity of the plastid ASVs, and the variance between the two populations is high (*R* ^2^ ~ .2 from the PERMANOVA, Table 2).

Our results show a differentiation of the microbiota composition between populations and among reproductive stages. One explanation for these trends is the differentiation of the diet, which has been demonstrated to be the main factor shaping the functionality and diversity of the gut microbiome, resulting in the convergence between microbial communities and their hosts’ foraging habits (Muegge et al., 2011). There is evidence that the diet shapes the functionality, diversity, and relative abundance of dominant phyla, as well as the populations of specific bacterial groups; this is influenced in part by the composition of macronutrients consumed and in part by the introduction of new microbes from food itself (Muegge et al., 2011; Voreades, Kozil, & Weir, 2014).

In the two populations of *L. yerbabuenae,* our results show significant differences in the beta diversity of lactating and pregnant females, perhaps associated with the high energy, protein and calcium requirements during these life stages (Gaona et al., 2019; Speakman, 2008). Previous work has shown that the diversity of plants consumed by *L. yerbabueanae* is 2.4 times higher among pregnant and lactating females than among nonreproductive females and males (Riechers, Martínez‐Coronel, & Vidal, 2003). This increased foraging diversity is also reflected in our results, with a higher diversity of gut microbes in pregnant and lactating females in the central population, likely due to the diversified diet due to the physiological conditions of pregnancy and lactation and the high plant diversity in the central region (Figure 6).

The analysis of chloroplasts showed a difference between the two populations; while there was some overlap between the central population and Pacific population adults, the two populations tended to separate, with lower diversity in the Pacific (Figure 6).

## 5 CONCLUSION

The differential fecal microbiota composition of the two *L. yerbabuenae* populations, central and Pacific, which inhabit geographically disjunct ranges that differ in their availability of nectar and pollen, opens the door to exploring microbiome–bat relationships that may be influenced by natural selection. Both variation and stability are present, suggesting that it is not just the host, but the host–microbiome unit, the “holobiont,” which could be subject to natural selection and evolutionary processes (Cerqueda‐García & Falcón, 2016; Suzuki, 2017). This study suggests a differentiation between both populations' microbiota as a consequence of geographical separation and food resources availability. Diversity was highest in the central population, where there is also higher genetic diversity, perhaps due to the unrestricted availability of foraging resources. This is in contrast to the Pacific population, which had lower microbiome and genetic variability, probably because resources are only available seasonally. These resource availability interactions influence reproduction and population size, with increased genetic diversity, both in the host and in the microbiome. Interestingly, these two populations share a core microbiota of 539 ASVs accounting for 75% of the relative abundance, being the most abundant microbiota stable over evolutionary time, and these results may support the phylosymbiosis theory.

## CONFLICT OF INTERESTS

None declared.

## AUTHOR CONTRIBUTIONS

Osiris Gaona: Conceptualization; Formal analysis; Funding acquisition; Investigation; Methodology; Project administration; Supervision; Visualization; Writing‐original draft; Writing‐review & editing. Daniel Cerqueda‐Garcia: Data curation; Formal analysis; Investigation; Methodology; Software; Validation; Visualization; Writing‐original draft; Writing‐review & editing. Andres Moya: Validation; Writing‐review & editing. Carla Ximena Neri‐Barrios: Investigation; Visualization; Writing‐original draft; Writing‐review & editing. Luisa Falcón: Conceptualization; Funding acquisition; Resources; Supervision.

## ETHICS STATEMENT

Sampling procedures strictly followed the guidelines of the American Society of Mammalogists for capture, handling and care of mammals (Gardner, 1976; Sikes & Gannon, 2011). To minimize animal suffering and distress, each animal was processed by experts only, ensuring safe and effective handling. Samples were taken from wild bats that were released in the same area of capture, causing no apparent harm to individuals. *Leptonycteris yerbabuenae* is not subject to federal protection under Mexican law (NOM‐059‐SEMARNAT‐2010). All activities were approved and authorized under a scientific collection permit granted by the Mexican Subsecretary of the Environment and Natural Resources (SEMARNAT), number FAUT‐0231, SGPA/DGVS/05780/15 SEMARNAT. Laboratory sample processing activities fulfilled all biosafety standard requirements of the Ecology Institute of the Universidad Nacional Autónoma de México (UNAM), under the authorization from the Biosafety Commission of the Ecology Institute, UNAM.

## Figures and Tables

**Figure A2:**
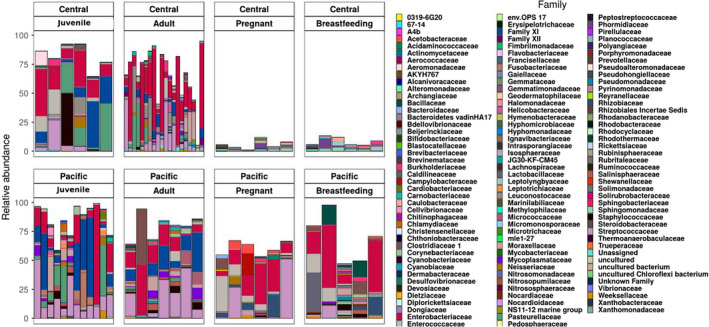
Complete shared families between populations

## Data Availability

The raw reads are available at the NCBI database under BioProjects PRJNA508738: https://www.ncbi.nlm.nih.gov/bioproject/PRJNA508738 and PRJNA543523: https://www.ncbi.nlm.nih.gov/bioproject/PRJNA543523.

## APPENDIX 1

**Table A1:** The number of reads of plastid 16S in each sample

Sample	Counts	Region	Stage
C11	1,386	Central	Adult
C13	331	Central	Adult
C15	551	Central	Adult
C17	1,421	Central	Adult
C19	13,395	Central	Adult
C21	3,747	Central	Adult
C23	12,756	Central	Adult
C26	2,881	Central	Juvenil
C27	10,388	Central	Juvenil
C28	5,026	Central	Juvenil
C29	913	Central	Juvenil
C3	732	Central	Adult
C30	59	Central	Juvenil
C32	57	Central	Juvenil
C33	24,607	Central	Adult
C34	15,030	Central	Adult
C35	6,045	Central	Adult
C36	5,791	Central	Adult
C41	3,075	Central	Adult
C42	7,323	Central	Adult
C43	16,467	Central	Adult
C44	14,362	Central	Adult
C5	997	Central	Adult
C57	184	Central	Lactating
C62	96	Central	Pregnant
C65	211	Central	Pregnant
C67	17	Central	Pregnant
C69	258	Central	Lactating
C7	603	Central	Adult
C71	780	Central	Pregnant
C73	126	Central	Lactating
C74	109	Central	Lactating
C76	30	Central	Adult
C77	322	Central	Lactating
C79	76,979	Central	Pregnant
C81	511	Central	Pregnant
C83	178	Central	Lactating
C9	1,412	Central	Adult
P1	1,204	Pacific	Lactating
P10	603	Pacific	Pregnant
P11	512	Pacific	Pregnant
P2	282	Pacific	Lactating
P20	146	Pacific	Juvenil
P21	1,366	Pacific	Juvenil
P22	1,425	Pacific	Adult
P23	40	Pacific	Adult
P24	27,831	Pacific	Juvenil
P25	47,656	Pacific	Adult
P27	4,636	Pacific	Adult
P28	1564	Pacific	Adult
P29	913	Pacific	Adult
P3	5,953	Pacific	Lactating
P30	7,114	Pacific	Juvenil
P31	39,840	Pacific	Juvenil
P32	420	Pacific	Juvenil
P33	179	Pacific	Juvenil
P34	736	Pacific	Adult
P35	181	Pacific	Juvenil
P36	90	Pacific	Juvenil
P37	334	Pacific	Juvenil
P38	299	Pacific	Juvenil
P39	1999	Pacific	Juvenil
P4	290	Pacific	Pregnant
P5	2093	Pacific	Lactating
P6	1907	Pacific	Pregnant
P7	362	Pacific	Pregnant
P8	432	Pacific	Pregnant
P9	978	Pacific	Lactating

## APPENDIX 2

### Extraction of DNA from feces

Metagenomic fecal DNA was extracted using the DNeasy Blood & Tissue kit (Qiagen) according to the manufacturer's instructions. Briefly, feces collected into 1.5 ml sterile tubes were diluted with 180 μl of ATL extraction buffer with 20 μl proteinase K (10 mg/ml). Tubes were mixed thoroughly by vortexing and were incubated at 56°C at 1,500 rpm for 50 min. 200 μl of AL Buffer with 200 μl ethanol (96%–100%) were added and mixed thoroughly by vortexing. The mixture was transferred into the DNeasy Mini spin column, washed with Buffer AW1 and then with AW2. The DNA was eluted with 200 μl of AE Buffer and precipitated with absolute ethanol, 0.1 volume 3 M sodium acetate and 2 µl GlycoBlue. DNA was resuspended in 30 µl of molecular grade water and stored at −20°C until PCR amplification.

### 16S rRNA gene amplification and sequencing

DNA samples were PCR‐amplified using the hypervariable V4 region of the 16S rRNA gene with universal bacterial/archaeal primers 515F/806R following the procedures reported by Caporaso et al. (2012) and Carrillo‐Araujo et al. (2015). PCR reactions (25 µl) contained 2–6 ng of total DNA, 2.5 µl Takara ExTaq PCR buffer 10X, 2 µl Takara dNTP mix (2.5 mM), 0.7 µl bovine serum albumin (BSA, 20 mg/ml), 1 µl primers (10 μM), 0.125 µl Takara Ex Taq DNA Polymerase (5 U/μl) (TaKaRa) and nuclease‐free water. Samples were amplified in triplicate using a PCR protocol consisting of an initial denaturation step at 95°C (3 min), followed by 35 cycles of 95°C (30 s), 52°C (40 s) and 72°C (90 s), followed by a final extension (72°C, 12 min). Triplicates were then pooled and purified using the SPRI magnetic bead, AgencourtAMPure XP PCR purification system (Beckman Coulter). The purified 16S rRNA fragments (~20 ng per sample) were sequenced on an IlluminaMiSeq platform (Yale Center for Genome Analysis), generating ~250 bp paired‐end reads.
